# Invasive plants decrease arbuscular mycorrhizal fungal diversity and promote generalist fungal partners

**DOI:** 10.1111/nph.70342

**Published:** 2025-06-26

**Authors:** John V. Ramana, Jason M. Tylianakis, Warwick J. Allen, Hayley J. Ridgway, Lauren P. Waller, Kate H. Orwin, Ian A. Dickie

**Affiliations:** ^1^ Bioprotection Aotearoa, School of Biological Sciences University of Canterbury Christchurch 8041 New Zealand; ^2^ Manaaki Whenua – Landcare Research Lincoln 7640 New Zealand; ^3^ The New Zealand Institute for Plant and Food Research Ltd Lincoln 7608 New Zealand; ^4^ Lincoln University Lincoln 7608 New Zealand

**Keywords:** AMF, exotic plants, invasion biology, plant invasion, root diameter, root traits

## Abstract

- Invasion by exotic plants is a major threat to ecosystem biodiversity globally. Although mutualistic belowground associations can play a significant role in successful invasion, studies have shown mixed results regarding the effects of plant invasion on arbuscular mycorrhizal fungi (AMF). Here, we tested how exotic dominance (i.e. invasion extent) in plant communities impacts arbuscular mycorrhizal fungal community diversity, composition, and generalism at the scale of individual plants and plant communities, and whether these impacts are explained or moderated by plant root traits (root diameter).
- We characterized root traits and arbuscular mycorrhizal fungal communities in the roots of native and exotic plants growing together in experimental plant communities that ranged in invasion extent (exotic plant dominance from 0% to 100%).
- Increases in exotic dominance decreased arbuscular mycorrhizal fungal diversity in both individual plant roots and plant communities. Greater relative abundance of generalist AMF was also observed in plant communities with increasing exotic dominance. Although root diameter affected fungal composition, it did not moderate or drive the effect of exotics.
- Our results highlight the role of invasion extent in understanding how arbuscular mycorrhizal fungal communities respond to exotic plant invasion and the importance of preserving belowground biodiversity.

## Introduction

Arbuscular mycorrhizal fungi (AMF) can facilitate or hinder the success of invasive exotic plants by modifying individual plant competitiveness and the diversity and structure of plant communities (Lin *et al*., 2015; Gerz *et al*., 2018; Aslani *et al*., 2019). The ‘degraded mutualisms’ hypothesis suggests that exotic plants have reduced associations with AMF in the invaded range and therefore may also reduce the diversity and abundance of available mutualists for native plants (Vogelsang & Bever, 2009). Although studies have shown that invasion by exotic plant species decreases arbuscular mycorrhizal fungal diversity in soils (Hawkes *et al*., 2006; Zubek *et al*., 2016; Řezáčová *et al*., 2021), other studies have shown the opposite (Lekberg *et al*., 2013; Gomes *et al*., 2018). For example, invasion by *Centaurea stoebe* and *Euphorbia esula* increased the diversity and abundance of AMF associating with native plants in Montana (USA), whereas *Bromus tectorum* had no effect (Lekberg *et al*., 2013). This variability suggests that the impacts of invasive plants on arbuscular mycorrhizal fungal diversity and community composition are species‐ and context‐specific. Studies to date have largely focused on specific plant invasions without accounting for the extent of invasion, potentially obscuring general patterns due to site‐ and species‐specific effects.

In other mutualistic interaction networks (e.g. plant–pollinator), successful invaders are typically generalist in their associations (Aizen *et al*., 2008) and show a weaker signal of trait matching due to a lack of coevolutionary history with their partners (Peralta *et al*., 2020). However, invasive plants can succeed in non‐native habitats despite a lack of coevolutionary history, by forming ‘familiar’ associations with widespread fungi (Moora *et al*., 2011; Dickie *et al*., 2017) that are often generalists in their interactions. Habitat generalist plants have been shown to frequently associate with generalist AMF (Öpik *et al*., 2009). For example, the arbuscular mycorrhizal fungal community of *Trachycarpus fortunei* (Chinese windmill palm) differed between its native and invaded range, although both populations shared a portion of widespread generalist AMF (Moora *et al*., 2011). In addition to generalist associations being a key mechanism by which exotic plants invade, in New Zealand grasslands, exotic plants have also been shown to associate with a lower diversity of AMF compared with native plants growing in the same location (Ramana *et al*., 2023). However, it is not well‐understood whether the loss in fungal diversity due to greater exotic dominance also promotes generalist fungal partners (fungi that are abundant and that also associate with many plants) in plant communities.

Community context can influence the composition of plant interaction partners because the traits of individual plants and their partner species must be compatible for the interaction to occur (i.e. trait matching). For example, plant root traits strongly influence both rhizosphere fungal and arbuscular mycorrhizal fungal communities: Sweeney *et al*. (2021) showed that the relative abundance of AMF in the rhizosphere of grassland plants increased with plant root diameter, while Lozano *et al*. (2021) found that root tissue density was an important driver of mycorrhizal abundance. Plant root diameter can also influence specificity in mycorrhizal symbioses, with fine‐rooted plants associating with more generalist AMF compared with coarse‐rooted plants (Ramana *et al*., 2023). These results indicate that plant species exhibit varying levels of partner specificity in their associations with AMF and that those with high host specificity (i.e. coarse‐rooted plants) may be most affected by changes in the fungal community. However, how root traits mediate the relationship between exotic plant dominance and AMF is not yet well‐understood.

To gain insights into how plant root traits and exotic dominance alter arbuscular mycorrhizal fungal diversity, composition, and specificity within plant communities, we characterized the arbuscular mycorrhizal fungal communities in the roots of native and exotic plants growing together in experimental eight‐species plant communities, ranging in exotic dominance from 0% to 100% (Waller *et al*., 2020). We examined the changes in arbuscular mycorrhizal fungal diversity and composition at both the individual plant and plant community scale, providing novel insights into how exotic plants interact with and modify mutualistic interactions in plant communities. Our hypotheses were as follows:

1. arbuscular mycorrhizal fungal diversity in both individual plants and plant communities will decrease as exotic numerical dominance of the community increases;
2. the relative abundance of generalist AMF will increase in plant communities as exotic dominance increases; and
3. the loss of fungal diversity caused by increasing exotic dominance will be greater in coarse‐rooted plants than in fine‐rooted plants.

## Materials and Methods

### Study site and experimental design

The experiment was initiated in 2016, in a field on the Lincoln University Campus (Lincoln, New Zealand, 43.6434:S, 172.4678:E, elevation 10 m), and completed in 2019 (Waller *et al*., 2020). Twenty plant communities were designed, each comprising eight plant species taken from a pool of 39 species (Supporting Information Table S1) and grown in 125‐l pots (575 mm diameter). The full methods are described in Waller *et al*. (2020). Briefly, the 20 plant communities varied in the proportion of exotic species that were planted (0%, 25%, 50%, 75%, and 100%). Each plant community was replicated eight times (160 total mesocosm pots). Each pot received one of two soil treatments (‘home’ or ‘away’ soil) made up of a mixture of soils from a monoculture plant–soil feedback experiment using a common field soil that was cultured by each of the plant species used in the experiment (Waller *et al*., 2020). Home soils contained a mixture of conditioned soils from each of the eight plant species occurring in that community, whereas away soils contained a mixture of conditioned soils from eight species that did not occur in that community but did occur in one of the other 19 communities. This inoculum was mixed with a pasteurized background sand/soil mixture. The soil treatment was fully crossed with a herbivore treatment, in which all mesocosms were caged and invertebrate herbivores were added or excluded (Allen *et al*., 2021). Herbivore populations were deliberately established in 80 mesocosms. Thirteen herbivore species that were added successfully established, along with seven self‐colonizing species, equaling a total of 20 different species. A detailed overview of the experimental design and methods (including the herbivore treatment and species) are supplied here in Methods S1.

### Root diameter measurements

For each plant species, we calculated the average diameter of each root order (1^st^, 2^nd^, 3^rd^) and took the mean of these measurement as the mean root diameter for each plant.

We used plants that were grown in pots in a glasshouse for 9 months. We subsampled a portion of the root of each plant that contained all possible root orders. We collected a single sample from at least three individuals of each species, which was stored in 70% ethanol before analysis. We fanned each root sample out on a Petri dish filled with water and measured the diameter at three random locations for each root order under a stereo microscope. There was no difference in average root diameter between native and exotic plants (*t*‐test *P =* 0.167, mean root diameter for exotic plants = 0.302 mm, and mean root diameter for native plants = 0.317 mm).

### Molecular sampling

DNA was extracted from a total of 982 roots that were harvested from individual plants from all 160 mesocosms at the end of the experiment (318 plants had died during the experiment), using MoBio (Carlsbad, CA, USA) PowerSoil extraction kits. Each plant was carefully excavated using a sterile spade. Plants were bagged in the field and then taken offsite to be washed free of soil and other debris. A small subsample of root was obtained from each individual plant by taking *c*. 10 fine‐root fragments from random places on the root ball. These were bundled together, and a 1‐cm cross section was cut from the bundle using a sterile razor blade. This sample was placed into a 96‐well plate from the MoBio Power Soil Extraction Kit and frozen at −80°C as soon as a plate was full. Libraries of the small subunit ribosomal RNA gene fragment amplicons were prepared using primers NS31 and AML2 with modifications to include the addition of technical adapter sequences for annealing to Illumina flow cells, a standard ‘pad’ sequence, and a two base pair linker sequence as in Morgan & Egerton‐Warburton (2017). The reverse primer (AML2) was also modified to include a 12‐base Golay barcode to enable demultiplexing during data processing. For each sample, PCR was carried out using 0.2 μl Taq (Roche FastStart) (Basel, Switzerland), 2.5 μl buffer, 0.5 μl dNTPs, 0.5 μl of 10 μM forward primer (NS31), 1 μl of 5 μM uniquely barcoded reverse primer (AML2), 0.45 μl of BSA, 18.85 μl of water, and 1 μl of genomic DNA extract, making up a 25 μl reaction volume.

The PCR was run using the following thermal cycler conditions: initial denaturation for 3 min at 94°C, followed by 45 cycles of 45 s at 94°C (denaturation), 60 s at 63.3°C (annealing), and 90 s at 72°C (extension); followed by a final extension step of 10 min at 72°C. PCR products were checked for adequate intensity single bands on a 1% agarose gel. PCR amplifications were carried out in duplicates for each sample and pooled before library preparation using the 96‐well SequalPrep Normalization Kit (Invitrogen, Waltham, MA, USA). Once PCR products were cleaned and normalized with the SequalPrep Kit, all samples were pooled and sent to Massey Genome Services to be sequenced on an Illumina MiSeq v.2, 2 × 250‐bp paired‐end chemistry (Illumina, San Diego, CA, USA).

### Bioinformatics and statistical analysis

We conducted all downstream analyses using only the 250‐bp forward reads as per Morgan & Egerton‐Warburton (2017). A study conducted by Davison *et al*. (2012) found that truncating NS31/AML2 reads from 400 to 170 bp resulted in near identical capture of AMF diversity as the majority of informative characters occurred in the 5′ most 170 base pairs. Sequences with less than 170 bp or which had more than one expected error were removed using Vsearch 2.10.4 (Rognes *et al*., 2016). To account for PCR and sequencing artifacts and singletons, any sequences occurring either once or twice were removed, and the remaining sequences were clustered to a 97% similarity threshold. Operational taxonomic units (OTUs) were matched using Blast v.2.5.0+ (Altschul *et al*., 1997) against the MaarjAM VT database (Öpik *et al*., 2010). We removed all OTUs that had < 200‐bp match and < 90% identity to any known species. Extraction blanks and negative controls were checked for contamination. OTUs that were found within negative controls were removed by subtracting the number of times that OTU appeared from all other samples. We removed all samples that had fewer than 1000 sequences, and excluded plants not known to associate with AMF from the analysis, resulting in the use of 692 root samples. A full list of plant species used in the analysis is available in Table S2. We also reran all analyses including all plant species (mycorrhizal and nonmycorrhizal) and found that the results did not change qualitatively.

We used R v.4.4.1 (R Development Core Team, 2019) for all downstream analyses and creating figures. For all arbuscular mycorrhizal fungal diversity calculations, we first generated randomly rarefied community versions of the dataset via the *rrarefy* function in vegan (Oksanen *et al*., 2001). The subsample size for rarefying the community was set to the minimum number of sequences in any sample. This process was iterated 500 times before calculating the average alpha, and gamma diversities (Schloss, 2023). The *rrarefy* function performs random rarefaction without replacement so that mean and variance of community metrics is not related to the size of the sample. Relationship between rarefied richness and number raw sequences is provided in Fig. S1.

### Individual plant‐level analyses

To quantify the effects of plant root diameter, plant provenance (i.e. native or exotic), and community context (i.e. proportion of exotics planted) on arbuscular mycorrhizal fungal diversity and relative abundance in individual plants, we used linear mixed effect models via the R package glmmtmb (Brooks *et al*., 2017). We performed model simplification using the function ‘dredge’ from the package mumin (Barton, 2012) to narrow down which predictor variables (if any) best predicted changes in arbuscular mycorrhizal fungal diversity and relative abundance. The model with the lowest degrees of freedom (i.e. most parsimonious) with a delta‐AIC (Akaike information criterion) of < 2 was selected as the best model for interpretation, and all statistically significant terms retained in the best model for each response variable are presented in the Results section.

For arbuscular mycorrhizal fungal richness in individual plant roots, we started with a maximal model including a three‐way interaction between root diameter, plant provenance, and proportion of exotics planted. Although the herbivore and soil treatments were not important to our hypotheses, we also included these as fixed terms in the model to account for the initial design of the experiment. We also included random terms for plant species and mesocosm nested within plant community. The same maximal model was used to test the relative abundance of different arbuscular mycorrhizal fungal families in individual plant roots. However, for those models, we used a zero‐inflated beta regression to analyze proportional data bounded between zero and one (Ferrari & Cribari‐Neto, 2004).

### Mesocosm/community‐level analyses

Gamma diversity was calculated as the total arbuscular mycorrhizal fungal richness of each mesocosm by aggregating the OTU table by mesocosm. When analyzing arbuscular mycorrhizal fungal gamma diversity at the mesocosm level, our maximal model had a two‐way interaction between the proportion of exotics planted and the average root diameter of all plants harvested from the mesocosm, with the herbivore treatment and soil treatment as fixed terms and with plant community and plant richness at harvest as random effects. We also included a fixed term for soil anaerobically mineralizable nitrogen (Curtin & Campbell, 2008) to account for any changes in soil fertility caused by the plant community. The maximal model was simplified using AIC model selection as described above, and all statistically significant terms retained in the best model for each response variable are presented in the Results section.

To evaluate whether plant communities with increasing exotic dominance had a higher relative abundance of generalist AMF, we calculated the exponent of Shannon diversity (*q* = 1) (Hill, 1973; Jost, 2006) for each OTU. This gives an indication of the ‘effective’ number of plant species each OTU interacted with. We then took the sum of the ‘effective number’ scores of all OTUs in each mesocosm and weighted by their proportional abundances as an overall ‘weighted generalism score’ for each mesocosm. We divided this score by the number of plant species present in each mesocosm at harvest to account for any biases caused by plant mortality. This metric reflects the degree of arbuscular mycorrhizal fungal generalism and abundance within the given plant community (the effective diversity of plant species in the experiment that each fungus is associated with and the fungal taxon's relative abundance when present) rather than an intrinsic ecological generalist–specialist tendency.

When analyzing generalism at the mesocosm level, our maximal model had a two‐way interaction between the proportion of exotics planted and the average root diameter of all plants harvested from the mesocosm, with the herbivore treatment and soil treatment as fixed terms and plant community as a random effect. The maximal model was simplified using AIC model selection as described previously, and all statistically significant terms retained in the best model for each response variable are presented in the Results section.

The summary statistics of all statistically significant results/selected models can be found in Notes S1–S7. All models were checked for homogeneity and normality of residuals, and overdispersion using the dharma package (Hartig, 2017). Assumptions were respected for all models except for the model testing the effects of root diameter and exotic dominance on the relative abundance of Archaeosporaceae, which had significant overdispersion. We therefore omitted this model from the results but have reported summary statistics in Notes S5. We also reran all analyses with proportion exotics realized at harvest and found that the results were not qualitatively different.

## Results

A total of 95 OTUs were obtained across 693 individual plants. An average of 15 455 reads were generated per sample (min = 1031 and max =165 978). The OTUs spanned four orders (Archaeosporales, Diversisporales, Glomerales, and Paraglomerales) and seven families (Acaulosporaceae, Archaeosporaceae, Calaroideoglomeraceae, Diversisporaceae, Gigasporaceae, Glomeraceae, and Paraglomeraceae). The three families with the highest OTU richness were Glomeraceae (43 OTUs), Acaulosporaceae (18 OTUs), and Archaeosporaceae (12 OTUs). A total of 10 703 706 reads were generated, of which 6075 054 (56.8%) reads were accounted for by the two most abundant OTUs, both of which belonged to the family Glomeraceae. Most OTUs were low dominance, with 84 of the 95 OTUs having maximum abundances of < 1% of sequences within any plant.

### Arbuscular mycorrhizal fungal richness

**Fig. 1:**
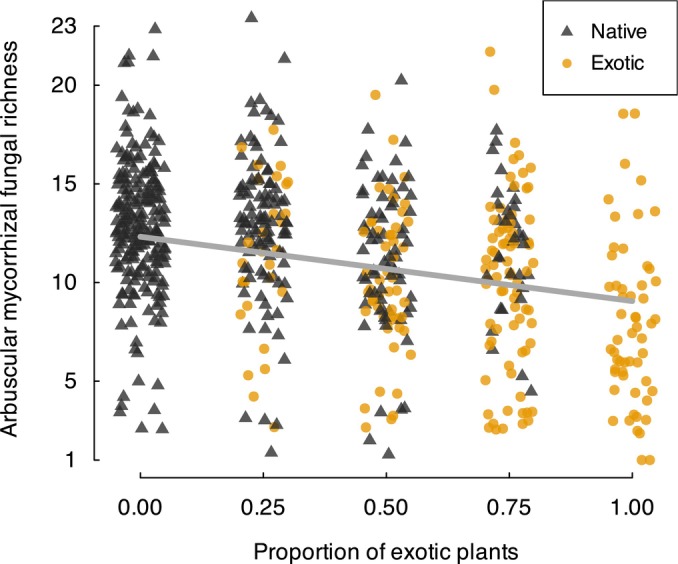
Richness of arbuscular mycorrhizal fungal operational taxonomic units (OTUs) from the roots of individual native (black triangles) and exotic (orange circles) plants within planted communities that varied in their proportion of exotic plant species. The trend line was fitted from the best model and shows the decline in arbuscular mycorrhizal fungal OTU richness with increasing exotic dominance of the plant community. Random noise was added to the *x*‐axis values to facilitate visualization of overlapping data points.

Arbuscular mycorrhizal fungal richness in plant roots decreased as the proportion of exotic plants planted in the community increased (*proportion exotic z* = −3.11, *P* = 0.002; Fig. 1), and this relationship did not depend on plant root diameter and provenance, or other community context predictors such as herbivore and soil treatments or their interaction.

### Relative abundance of arbuscular mycorrhizal fungal families

**Fig. 2:**
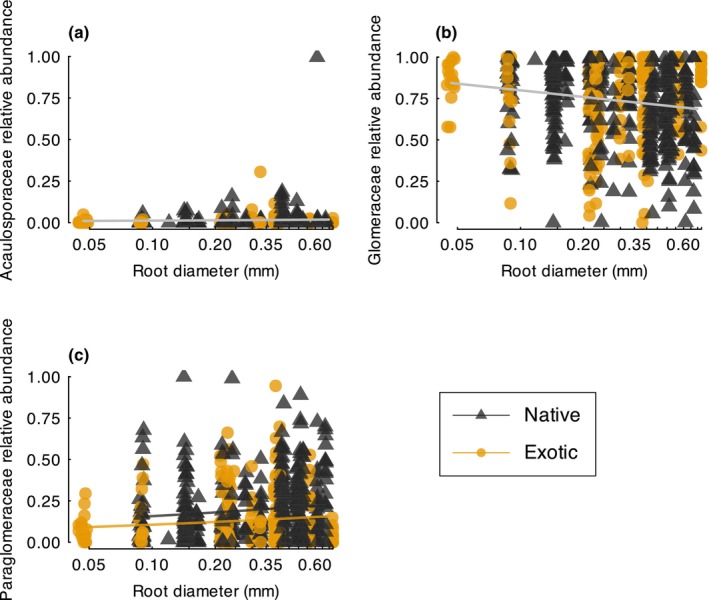
(a–c) Correlations between native (black triangles) and exotic (orange circles) plant root diameter and the relative abundance of arbuscular mycorrhizal fungi from the families Acaulasporaceae (a), Glomeraceae (b), and Paraglomeraceae (c). Trend lines were fitted from the best models. The black and orange lines show the trend for native and exotic plants, respectively Trend lines are shown in gray for models in which plant provenance was not a significant predictor. Random noise was added to the *x*‐axis values to facilitate visualization of overlapping data points.

Changes in the composition of AMF in plant roots was correlated with root diameter and plant provenance. The relative abundance of three arbuscular mycorrhizal fungal families showed a significant response to plant root diameter. The relative abundance of Acaulosporaceae and Glomeraceae showed contrasting positive (*root diameter*, *z* = 2.38, *P* = 0.017; Fig. 2a) and negative (*root diameter*, *z* = −2.17, *P* = 0.030; Fig. 2b) correlations with plant root diameter, respectively. The relative abundance of AMF from the family Paraglomeraceae increased in coarse‐rooted plants (*root diameter* + plant provenance, *z* = 2.37, *P* = 0.018; Fig. 2c) and was higher for native than for exotic plants (root diameter + *plant provenance*, *z* = 3.27, *P* = 0.001; Fig. 2c).

### Gamma diversity

**Fig. 3:**
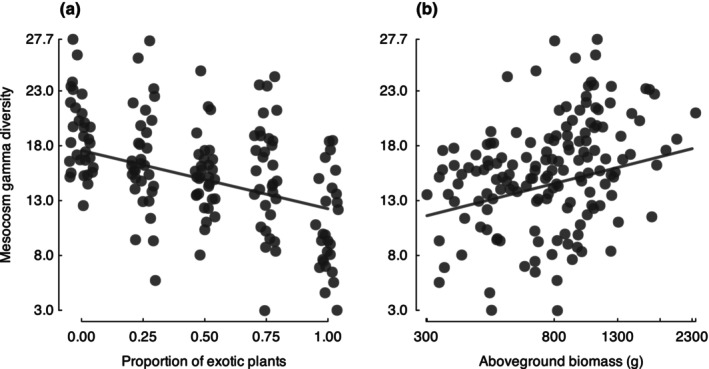
Relationships between gamma diversity (at the community level) of arbuscular mycorrhizal fungal operational taxonomic units and (a) the proportion of exotic plants planted in a mesocosm community and (b) the aboveground biomass of plant communities. Trend lines were fitted from the best model. Random noise was added to the *x*‐axis of panel a to facilitate visualization of overlapping data points.

When data were aggregated at the community level, the gamma diversity of AMF was best predicted by the proportion of exotics planted, aboveground biomass, and the herbivore treatment. Arbuscular mycorrhizal fungal gamma diversity decreased as the proportion of exotics planted in the community increased (*proportion exotic* + aboveground biomass + herbivores: *z* = −2.54, *P* = 0.01; Fig. 3a). There was also a positive relationship between aboveground biomass and arbuscular mycorrhizal fungal gamma diversity (proportion exotic + aboveground biomass + herbivores: *z* = 3.02, *P* = 0.003; Fig. 3b); however, aboveground biomass was not significantly correlated with the proportion of exotics planted. Herbivore addition reduced gamma diversity of AMF (proportion exotic + aboveground biomass + *herbivores*: *z* = 2.46, *P* = 0.02).

### Generalism of fungal partners

**Fig. 4:**
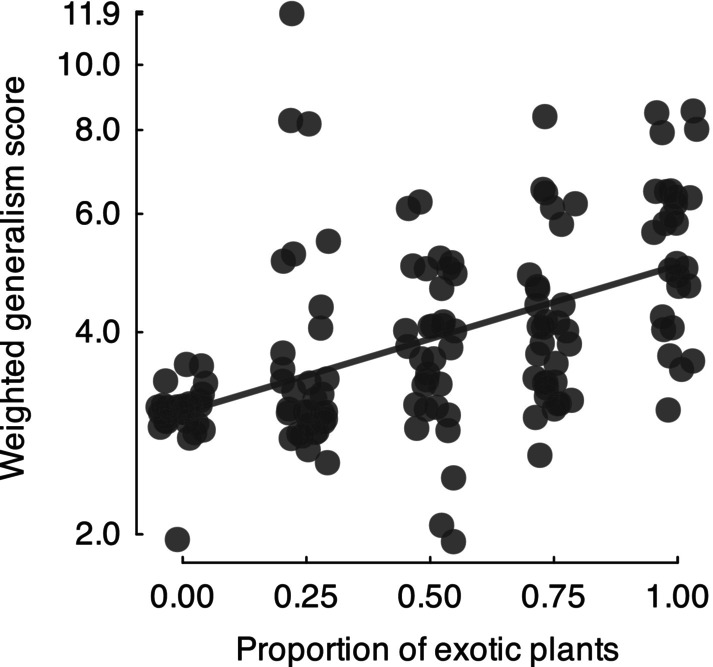
Relationship between the weighted generalism score of mesocosm plant communities and the proportion of exotic plants that were planted in a mesocosm community. The trend line was fitted from the best model. Random noise was added to the *x*‐axis values, and weighted generalism score values have been log‐transformed to facilitate visualization of overlapping data points.

Exotic dominance in plant communities led to an increase in generalist fungal partners. The weighted generalism scores increased with the proportion of exotic plants that were planted (*proportion exotic*, *z* = 3.80, *P* < 0.001; Fig. 4).

## Discussion

Previous studies have found mixed results concerning the impact of exotic plants on the diversity and composition of arbuscular mycorrhizal fungal communities (Hawkes *et al*., 2006;Gomes *et al*., 2018; Řezáčová *et al*., 2021). Here, we show how plant root traits and the extent of exotic invasion can influence mycorrhizal fungal associations within individual plant roots and in plant communities. Exotic plant dominance reduced arbuscular mycorrhizal fungal diversity in the roots of individual plants and their communities, while increasing the relative abundance of generalist fungal partners. Associating with widespread generalist mutualistic partners could be a strategy that supports the successful establishment of exotic plants (Dickie *et al*., 2017).

### Exotic plants reduced arbuscular mycorrhizal fungal diversity in plant roots

Invasion by exotic plant species has previously been shown to have mixed effects on the diversity of AMF in the soil (Gomes *et al*., 2018; Řezáčová *et al*., 2021). Here, by manipulating invasion extent and using a wide range of plant species, we found that arbuscular mycorrhizal fungal richness decreased in both native and exotic plants as the dominance of exotic plants in the community increased, providing some support for the degraded mutualisms hypothesis (Vogelsang & Bever, 2009). The degraded mutualisms hypothesis suggests that invasive plants have reduced associations and dependency on AMF, which may decrease arbuscular mycorrhizal fungal abundance and/or richness. A mechanism by which exotic plants may reduce mycorrhizal abundance is by increasing soil nutrient availability and reducing plant dependency on mycorrhizas (Grove *et al*., 2017); however, in our case, soil nitrogen was not a strong driver of mycorrhizal fungal diversity or community composition. In the same experiment, Waller *et al*. (2020) found that exotic plants increased the abundance of gram‐negative bacteria, which can be antagonistic toward arbuscular mycorrhizal fungal hyphae (Svenningsen *et al*., 2018). This could be a mechanism by which specialist or low abundance taxa are lost causing a decrease in fungal diversity. A decrease in the richness of AMF may also decrease the functional diversity of mycorrhizal partners with which native plants may typically engage, perhaps facilitating invasion of exotic species.

In contrast to the degraded mutualisms hypothesis, there is also evidence showing that exotic plants have enhanced mutualisms with fungal partners (Zhang *et al*., 2010; Lekberg & Callaway, 2022; Yu *et al*., 2022). For example, when comparing 19 pairs of congeneric plant species, Yu *et al*. (2022) showed that, relative to natives, invasive plants had greater arbuscular mycorrhizal colonization and plant biomass, and plant root exudates from invasive plants increase arbuscular mycorrhizal fungal infection. While differences in root exudation could influence interactions with AMF, the strength of fungal response could depend on fungal species identity (Zhang *et al*., 2010). However, examining plants singularly or within pairs may not be reflective of the full breadth of interactions that may take place in complex plant communities and therefore may have led to results that contrast with those of our study.

### Exotic plant dominance promoted generalist arbuscular mycorrhizal fungi

Exotic species in many plant–mutualist networks, including plant–mycorrhizal interactions, have been documented to associate with generalist partners (Aizen *et al*., 2008; Bunn *et al*., 2014; Weber *et al*., 2019). Exotic plants have been shown to harbor a greater relative abundance of generalist pathogens compared with native plants (Waller *et al*., 2024). However, whether exotic plants promote generalist mutualistic associations in plant communities was previously unclear. In support of our second hypothesis, communities with high exotic dominance associated more with generalist arbuscular mycorrhizal fungal partners than those with low exotic dominance. Our results complement the findings of Waller *et al*. (2020), who in the same experiment, showed a negative correlation between arbuscular mycorrhizal fungal biomass and exotic dominance, suggesting a reduction in C allocation to fungal partners. Reduction in C allocation to fungi could result in less abundant fungi that may be more host‐specific being outcompeted by widespread generalist fungi. This shift in the composition of mutualists may facilitate the spread and persistence of exotic plants by reinforcing associations with AMF that are generalists (Rodríguez‐Caballero *et al*., 2018).

### Root traits and plant provenance influenced arbuscular mycorrhizal fungal community composition

The loss of fungal diversity caused by exotic dominance was not different between coarse and fine‐rooted plants; however, we found that root diameter was correlated with the relative abundance of multiple arbuscular mycorrhizal fungal families for both native and exotic plants. This supports previous work in New Zealand grasslands, indicating that both root diameter and plant provenance can influence arbuscular mycorrhizal fungal community composition (Ramana *et al*., 2023). Although exotic dominance had a significant effect on the richness of AMF, it did not influence the relative abundance of any arbuscular mycorrhizal fungal families. Furthermore, Day *et al*. (2015) showed that arbuscular mycorrhizal fungal communities colonizing an invasive plant grown in previously uninvaded soil did not change to resemble those growing in invaded soils. These results suggest that changes in arbuscular mycorrhizal fungal community composition as a result of invasion may unfold slowly over time (Mummey & Rillig, 2006; Day *et al*., 2015). Moreover, a meta‐analysis of 67 studies by Bunn *et al*. (2015) reported no change in arbuscular mycorrhizal fungal abundance between native and exotic plants, although their study did not incorporate plant root traits. We did find that exotic plants had a significantly lower relative abundance of AMF from the family Paraglomeraceae compared with native plants. Fungi of this family are typically known to have high investment in intraradical hyphae and low extraradical hyphal abundance (Phillips *et al*., 2019). This indicates that exotic species may associate with fungi that explore the large soil volume and are therefore more likely to be encountered by other plants. This further supports the patterns of increasing fungal generalism with exotic dominance in our study.

### Exotic dominance reduced arbuscular mycorrhizal fungal diversity in plant communities

Invasion by exotic plants can alter belowground interactions (Zubek *et al*., 2016; Gaggini *et al*., 2019; Sapsford *et al*., 2022), plant diversity (Hejda *et al*., 2009), and hinder plant growth. Our experimental design allowed us to not only show the impact exotic plants have belowground but also observe shifts in fungal communities that coincided with experimental shifts in plant communities (i.e. aboveground biomass). The observed positive correlation between mesocosm fungal gamma diversity and aboveground biomass suggests that the loss of diversity caused by exotic plants has a negative impact on plant communities aboveground. The loss of mycorrhizal fungal diversity at both the individual and community scale could be linked to a loss of functional diversity (Li *et al*., 2022; Renault *et al*., 2022) in belowground partners, potentially hindering plant growth. Greater arbuscular mycorrhizal fungal diversity has been linked to an increase in plant biomass and higher phosphorus acquisition alongside lower C allocation to AMF (Weber *et al*., 2025). Therefore, having access to a range of fungal partners may allow for more favorable resource exchange, supporting the increase in aboveground biomass seen in communities with greater fungal diversity. The presence of other microbes and/or pathogens that may have survived soil pasteurization could have also influenced plant growth and plant–fungal interactions. However, the use of live home and away soil inoculum may have helped to reduce the impact of low‐level background microbes as significant changes in fungal diversity and composition based on plant and plant community context were still evident. We also found that mesocosms without herbivores had a higher diversity of AMF. Herbivory by aphids has been shown to reduce carbon supply to extraradical mycorrhizal fungal hyphae (Charters *et al*., 2020; Durant *et al*., 2023), suggesting that the loss in fungal diversity could be due to a herbivore‐induced reduction in carbon allocation to mycorrhizal fungi. Links between herbivory and arbuscular mycorrhizal fungal diversity have previously been reviewed by Frew *et al*. (2022).

### Conclusions

Invasion by exotic plants is a major threat to ecosystems globally. Examining the role that AMF play at both the plant and community level is paramount to developing novel approaches to manage and minimize the impacts of exotic plant invasion. We found that exotic plant dominance decreased the diversity of AMF and promoted generalist fungal partners in plant communities. The loss of arbuscular mycorrhizal fungal diversity was also correlated with a decline in aboveground biomass. Therefore, arbuscular mycorrhizal fungal diversity can be an important indicator of successful ecosystem restoration.

## Competing interests

None declared.

## Author contributions

IAD and JMT obtained funding. IAD, JMT, LPW and WJA designed the original experiment. LPW and WJA implemented the original experiment and collected data. JVR processed the AM fungal samples, conducted analyses and wrote the first draft of the manuscript under the mentorship of IAD, JMT, HJR and KHO. All authors contributed substantially to revisions.

## Disclaimer

The New Phytologist Foundation remains neutral with regard to jurisdictional claims in maps and in any institutional affiliations.

## Supporting information


**Fig. S1** Relationship between rarefied richness and the number of raw sequences.
**Methods S1** Methods adapted from the supplementary material of Waller *et al*. (2020).
**Notes S1** Model summary for arbuscular mycorrhizal richness in individual plant roots.
**Notes S2** Model summary for the relative abundance of Acaulosporaceae in individual plant roots.
**Notes S3** Model summary for the relative abundance of Glomeraceae in individual plant roots.
**Notes S4** Model summary for the relative abundance of Paraglomeraceae in individual plant roots.
**Notes S5** Model summary for the relative abundance of Archaeosporaceae in individual plant roots.
**Notes S6** Model summary for the gamma diversity of arbuscular mycorrhizal fungi in plant communities.
**Notes S7** Model summary for the weighted generalism score in plant communities.
**Table S1** List of all plant species used in the initial experiment and their provenance, functional group, mycorrhizal status, and nitrogen fixation status.
**Table S2** Subset of the original list of plant species known to associate with arbuscular mycorrhizal fungi and used in our analysis, along with each plant species provenance, functional group, nitrogen fixation status, and 1st‐, 2nd‐, and 3rd‐order root diameters.
Please note: Wiley is not responsible for the content or functionality of any Supporting Information supplied by the authors. Any queries (other than missing material) should be directed to the *New Phytologist* Central Office.

## Data Availability

All data supporting the findings of this study, including sequences and accession numbers, can be found on doi: 10.5281/zenodo.15621134.
