# Supplementary material for: Invasive plants decrease arbuscular mycorrhizal fungal diversity and promote generalist fungal partners
Source: New Phytol. 2025 Jun 26;247(5):2381–9. doi: 10.1111/nph.70342 (PMC12329189; doi:10.1111/nph.70342)
Supplement: Supplementary file 1 — Fig. S1 Relationship between rarefied richness and the number of raw sequences. Methods S1 Methods adapted from the supplementary material of Waller et al. (2020). Notes S1 Model summary for arbuscular mycorrhizal richness in individual plant roots. Notes S2 Model summary for the relative abundance of Acaulosporaceae in individual plant roots. Notes S3 Model summary for the relative abundance of Glomeraceae in individual plant roots. Notes S4 Model summary for the relative abundance of Paraglomeraceae in individual plant roots. Notes S5 Model summary for the relative abundance of Archaeosporaceae in individual plant roots. Notes S6 Model summary for the gamma diversity of arbuscular mycorrhizal fungi in plant communities. Notes S7 Model summary for the weighted generalism score in plant communities. Table S1 List of all plant species used in the initial experiment and their provenance, functional group, mycorrhizal status, and nitrogen fixation status. Table S2 Subset of the original list of plant species known to associate with arbuscular mycorrhizal fungi and used in our analysis, along with each plant species provenance, functional group, nitrogen fixation status, and 1st‐, 2nd‐, and 3rd‐order root diameters. Please note: Wiley is not responsible for the content or functionality of any Supporting Information supplied by the authors. Any queries (other than missing material) should be directed to the New Phytologist Central Office. [file NPH-247-2381-s001.pdf]

New Phytologist

***New Phytologist* Supporting Information**

Article title: Invasive plants decrease arbuscular mycorrhizal fungal diversity and promote generalist fungal partners
Authors: John V. Ramana, Jason M. Tylianakis, Warwick J. Allen, Hayley J. Ridgway, Lauren P. Waller, Kate H. Orwin, Ian A. Dickie
Article acceptance date: 10 June 2025

The following Supporting Information is available for this article:

Methods S1 Methods adapted from the supplementary material of Waller et al. (2020). *Seed collection and germination* Seeds of experimental plant species were collected from sites across a wide geographical area, from Molesworth Station in the Marlborough region in the north, Arthur's Pass to the south and west, and Banks Peninsula to the south and east. Seeds of 19 native and 20 exotic species were collected in paper bags and stored at 4◦C (see table S2 for list of species). Seeds we were unable to collect were ordered from NZSeeds, Proseeds and Southern Woods Nursery. Species that required stratification were added to a sterile potting medium (50:50 vermiculite and perlite) and refrigerated for 4-6 weeks. After removal from cold storage, they were placed in germination trays in the glasshouse and kept moist until germination. Species varied in their stratification period and time to germination. Some species we were unable to propagate, so we collected cuttings and rooted them in trays containing the perlite-vermiculite mix. *Soil conditioning* The soils to be used in the larger mesocosm experiment were initially conditioned in the glasshouse by each of our focal species in order to create unique 1) ‘home’ soil inocula that would contain the suite of specialist and generalist soil biota cultured by the resident species growing in a given community and 2) ‘away’ soil inocula that would contain soil organisms not typically associated with the resident plants in a

community. These soils could be practically interpreted as ‘previously-invaded’ vs. ‘uninvaded’. We grew 12-20 replicates of each individual plant species in 10 L pots containing live, field-collected soil mixed in equal parts with pasteurized field-collected soil and pasteurized sand. The soil was collected in late June and early July of 2016 from twelve subalpine, grass and shrub dominated sites located across western Canterbury and southern Marlborough. We chose sites where subsets of our focal plant species (table S1) were present. At each site, we located three sampling areas that were representative of the site, but differed slightly in species composition, so that our final soil sample included rhizosphere soil from as many of our focal species as possible. At each sampling area, we used shovels to excavate chunks of sod measuring approximately 1 x 0.5 x 0.2 m, retaining as much loose soil that had fallen off the sod as possible. Soil and sod were stored outdoors for two weeks in weatherproof bags where temperatures averaged 1/11° C (low/high). Aboveground vegetation was removed from sod chunks and soil and roots were passed through (2.5 cm^{2}) sieves in mid-July to remove rocks and other debris, but retain any roots. This sampling provided us with approximately 375 L of soil from each site, which was mixed together in equal parts for a total of approximately 3000 L of inoculum. We also collected 3000 L of soil from a field on the Lincoln University campus for pasteurization in mid-July of 2016. Sand was collected from the Rakaia River valley, washed and sieved. The soil collected from the Lincoln site was mixed with the sand in equal parts and pasteurized in 500 L batches on a modified trailer bed fitted with steam pipes below a metal sheet and covered with a large tarpaulin. Each batch was brought up to a temperature of at least 100°C, held at temperature for 60 minutes and cooled for 24 hours, before being treated a second time. Live soil was mixed with pasteurized soil in a 1:2 ratio. After seeds had germinated

and seedlings had at least one true leaf, they were transplanted into their own 1 L pot containing the soil inoculum. Some seedlings were too small to transplant directly into the large pot at this stage, so they were potted into a 500 mL pot of live inoculum until they were strong enough to go into the larger pot. Plants were added to pots beginning in July 2016 and grew for approximately 9-10 months. *Mesocosm experimental design* Our mesocosm experiment incorporated a fully factorial design, with 20 plant communities × soil manipulation (home/away) × herbivore manipulation (herbivores added/not added). To begin, we designed 20 unique plant communities, each containing one individual of each of eight species, varying orthogonally in their proportion of exotic species (0, 25, 50, 75, 100%) and their proportion of woody species (0, 25, 38, 63%). Although we initially included seven nitrogen (N)-fixing exotic species, two species (one woody and one herbaceous) had extremely low survival, so effectively there were only five exotic N-fixers overall. Home soils contained a mixture of conditioned soils from each of the eight species occurring in that community, whereas away soils contained a mixture of conditioned soils from eight species occurring in one of the other 19 communities, but where a focal species did not occur. Twenty herbivore species established across the +Herbivore mesocosms (n = 80) and mesh cages were secured over each pot to ensure the insects remained in their pots. Seven of these herbivore species colonized from outside of cages (i.e. slugs and aphids) and we controlled these additions in -Herbivore but not +Herbivore mesocosms, where we allowed them to maintain populations. Thus, we consider our herbivore treatment to be ‘herbivores added’ vs. ‘herbivores reduced’. *Mesocosm establishment* The 160 mesocosms were established in a field on the campus of Lincoln University in Lincoln, New Zealand. We established the mesocosm communities in two phases. First, we germinated all of

our plant species a second time, using the same method as described above. This time, however, after germination and two true leaves emerged, we planted each of our species into small pots containing their respective mesocosm soil inoculum before adding them to the mesocosms. This was done so the plants could be "hardened off" in treated soil before planting outdoors. To accomplish this, we harvested four pots of each plant species grown in the soil-conditioning phase, combining all soil and roots into a single bag for each individual species. We simultaneously pasteurized another batch of field-collected background soil from our field at Lincoln University to double this inoculum volume. Each seedling for the mesocosm phase was then allocated to a particular community and treatment combination: plants that were to go into home soil communities were planted in individual pots in soil conditioned by themselves, and seedlings to grow in away communities were planted in soil from one randomly chosen species from their predetermined away mixture. These seedlings grew for approximately one month and were moved outside to a shade house during their last week in the small pots. While seedlings were hardening off, we harvested the rest of the plants from the conditioning phase as before and created home and away soil inoculum mixtures that would go into each mesocosm pot. Finally, to complete the planting, we constructed steel pots (575 mm diameter), filled each with a bottom layer of 22 L of crushed gravel, then 88 L of pasteurized soil:sand, and finally 12 L of either home or away soil inoculum, mixed uniformly across the top. We mixed pasteurized soil with sand to improve the drainage in the soil. Seedlings were planted in a ring, equally spaced around the center of the pot in March of 2017. *Mesocosm harvest and sampling*

After one year of growth, we harvested all above and belowground plant material from each mesocosm community. Using spades, we carefully excavated each plant, disentangling roots of

different species when necessary. Plants were bagged in the field and taken offsite to wash free of soil and other debris, then cut into root and shoot fractions. All shovels used for harvesting were scrubbed and rinsed in bleach for at least 10 minutes between mesocosms. Although the communities grew in pots for a year, there was no evidence that the plants were pot bound at the end of the experiment. The different plant species' roots were easily separated from one another at harvest, and there was no root coiling around the pots whatsoever. After all plants were removed, soil was homogenized by turning over for several minutes with the shovel, after which time a soil sample was extracted and taken into the laboratory. There, approximately 50 g of soil was passed through a 4 mm sieve, and stored at 4◦C until processed (i.e. substrate induced respiration, basal respiration). Ten grams of soil was freeze dried for PLFA/NLFA analysis, 50 g dried and ground for nutrient analyses, 2.5 g added to 96 well plates from the Mo-Bio Power Soil Extraction Kit, and 10 g frozen at -80$_o$C for archiving. In the laboratory, a small subsample of root was taken from each individual plant by taking approximately ten fine-root fragments from random places on the root ball, which were then bundled together, and a 1 cm cross section cut from the bundle using a sterile razor blade. This sample was placed into a 96 well plate from the Mo-Bio Power Soil Extraction Kit and frozen at -80$_o$C as soon as a plate was full. After this sampling, all plant material was dried at 65◦C and weighed. <u>Visual identification of mycorrhizae in plant roots</u> In order to assign mycorrhizal identification to some plant species. Plants were grown in pots inside a greenhouse and harvested after 6 months. Roots were stained according to (Kowal *et al.*, 2020). Roots were examined under a microscope for arbuscules, vesicles, and hyphae across 50 intersections from three different plants (150 intersections per plant). Plants were deemed associating with AMF if the presence of at least 2

of the tree structures were present. This was only for the basis of mycorrhizal type classification.

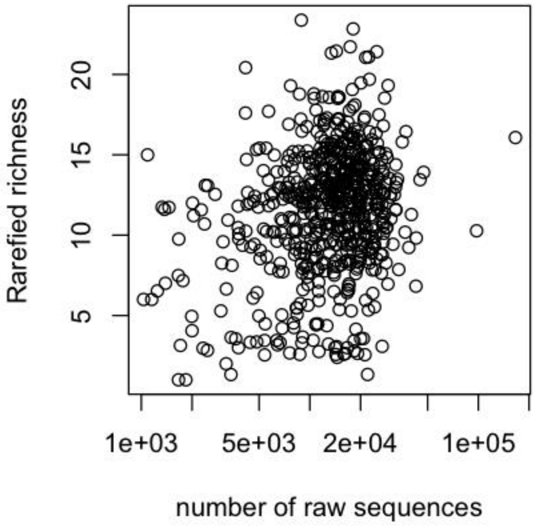

Fig. S1 showing the relationship between rarefied richness and the number of raw sequences

New Phytologist

Notes S1 Model summary for arbuscular mycorrhizal richness in individual plant roots

```
Family: gaussian  ( identity )
Formula:          alpha ~ pexoticVector + (1 | commVector/mesoVector) + (1
| speciesVector)
Data: runData1
     AIC      BIC   logLik deviance df.resid
  3283.6   3310.3  -1635.8   3271.6      623
Random effects:
Conditional model:
 Groups                Name        Variance Std.Dev.
 mesoVector:commVector (Intercept) 2.073    1.440
 commVector            (Intercept) 1.723    1.313
 speciesVector         (Intercept) 1.704    1.305
 Residual                          8.038    2.835
Number of obs: 629, groups:  mesoVector:commVector, 155; commVector, 20;
speciesVector, 30
Dispersion estimate for gaussian family (sigma^2): 8.04
Conditional model:
              Estimate Std. Error z value Pr(>|z|)
(Intercept)    12.3205     0.6554  18.800   <2e-16 ***
pexoticVector  -3.2626     1.0506  -3.106   0.0019 **
Signif. codes:  0 '***' 0.001 '**' 0.01 '*' 0.05 '.' 0.1 ' ' 1
```

New Phytologist

Notes S2 Model summary for the relative abundance of Acaulosporaceae in individual plant roots

```
Family: beta  ( logit )
Formula:          acuabund ~ log(rootDVector) + (1 | commVector/mesoVector) +
(1 |      speciesVector)
Zero inflation:            ~1
Data: runData1
     AIC      BIC   logLik deviance df.resid
 -1092.3  -1061.2    553.1  -1106.3      622
Random effects:
Conditional model:
 Groups                Name        Variance  Std.Dev.
 mesoVector:commVector (Intercept) 2.072e-11 4.552e-06
 commVector            (Intercept) 3.175e-11 5.635e-06
 speciesVector         (Intercept) 3.488e-09 5.906e-05
Number of obs: 629, groups:  mesoVector:commVector, 155; commVector, 20;
speciesVector, 30
Dispersion parameter for beta family (): 8.77
Conditional model:
                 Estimate Std. Error z value Pr(>|z|)
(Intercept)      -3.12132    0.15094 -20.680   <2e-16 ***
log(rootDVector)  0.21849    0.09331   2.341   0.0192 *
Signif. codes:  0 '***' 0.001 '**' 0.01 '*' 0.05 '.' 0.1 ' ' 1
```

Notes S3 Model summary for the relative abundance of Glomeraceae in individual plant roots

```
Family: gaussian  ( identity )
Formula:          gloabund ~ log(rootDVector) + (1 | commVector/mesoVector) + 
(1 |      speciesVector)
Data: runData1
     AIC      BIC   logLik deviance df.resid 
  -155.8   -129.1     83.9   -167.8      623 
Random effects:
Conditional model:
 Groups                Name        Variance Std.Dev.
 mesoVector:commVector (Intercept) 0.005378 0.07333 
 commVector            (Intercept) 0.002612 0.05111 
 speciesVector         (Intercept) 0.006312 0.07945 
 Residual                          0.036760 0.19173 
Number of obs: 629, groups:  mesoVector:commVector, 155; commVector, 20; 
speciesVector, 30
Dispersion estimate for gaussian family (sigma^2): 0.0368 
Conditional model:
                 Estimate Std. Error z value Pr(>|z|)    
(Intercept)       0.66613    0.04051  16.445   <2e-16 ***
log(rootDVector) -0.05790    0.02662  -2.175   0.0296 *  
Signif. codes:  0 '***' 0.001 '**' 0.01 '*' 0.05 '.' 0.1 ' ' 1
```

Notes S4 Model summary for the relative abundance of Paraglomeraceae in individual plant roots

```
Family: beta  ( logit )
Formula:          parabund ~ log(rootDVector) + nativeExoticVector + (1 |
commVector/mesoVector) +      (1 | speciesVector)
Zero inflation:            ~1
Data: runData1

     AIC      BIC   logLik deviance df.resid
  -469.5   -434.0    242.8   -485.5      621
Random effects:
Conditional model:
 Groups                Name        Variance Std.Dev.
 mesoVector:commVector (Intercept) 0.06011  0.2452
 commVector            (Intercept) 0.03291  0.1814
 speciesVector         (Intercept) 0.05623  0.2371
Number of obs: 629, groups:  mesoVector:commVector, 155; commVector, 20;
speciesVector, 30
Dispersion parameter for beta family (): 3.98
Conditional model:
                         Estimate Std. Error z value Pr(>|z|)
(Intercept)              -1.48761    0.17743  -8.384  < 2e-16 ***
log(rootDVector)          0.23575    0.09898   2.382  0.01723 *
nativeExoticVectorNative  0.46064    0.14083   3.271  0.00107 **
Signif. codes:  0 '***' 0.001 '**' 0.01 '*' 0.05 '.' 0.1 ' ' 1
```

Notes S5 Model summary for the relative abundance of Archaeosporaceae in individual plant roots

```
Family: beta  ( logit )
Formula:          (arcabund) ~ pexoticVector + (1 | commVector/mesoVector) +
(1 |      speciesVector)
Zero inflation:              ~1
Data: runData1

     AIC      BIC   logLik deviance df.resid
 -1398.2  -1367.1    706.1  -1412.2      622
Random effects:
Conditional model:
 Groups                Name        Variance  Std.Dev.
 mesoVector:commVector (Intercept) 2.039e-09 4.516e-05
 commVector            (Intercept) 1.762e-02 1.327e-01
 speciesVector         (Intercept) 1.334e-01 3.652e-01
Number of obs: 629, groups:  mesoVector:commVector, 155; commVector, 20;
speciesVector, 30
Dispersion parameter for beta family (): 8.83
Conditional model:
              Estimate Std. Error z value Pr(>|z|)
(Intercept)    -3.6816     0.1679 -21.927  < 2e-16 ***
pexoticVector   0.6892     0.2302   2.993  0.00276 **
Signif. codes:  0 '***' 0.001 '**' 0.01 '*' 0.05 '.' 0.1 ' ' 1

---
```

Notes S6 Model summary for the gamma diversity of arbuscular mycorrhizal fungi in plant communities

```
Family: gaussian  ( identity )
Formula:          gamma ~ Herbivore + log(mesoabVector) + prop.exotic + (1 |
Community) +      (1 | Real.richness)
Data: mesoMeta
     AIC      BIC   logLik deviance df.resid
   856.8    878.1   -421.4    842.8      148
Random effects:
Conditional model:
 Groups        Name        Variance Std.Dev.
 Community     (Intercept)  3.793   1.948
 Real.richness (Intercept)  2.312   1.521
 Residual                  10.707   3.272
Number of obs: 155, groups:  Community, 20; Real.richness, 7
Dispersion estimate for gaussian family (sigma^2): 10.7
Conditional model:
                  Estimate Std. Error z value Pr(>|z|)
(Intercept)        0.08173    5.78498   0.014  0.98873
HerbivoreNO_HERB   1.22685    0.54614   2.246  0.02468 *
log(mesoabVector)  2.49334    0.82496   3.022  0.00251 **
prop.exotic       -4.77139    1.87607  -2.543  0.01098 *
Signif. codes:  0 '***' 0.001 '**' 0.01 '*' 0.05 '.' 0.1 ' ' 1
```

Notes S7 Model summary for the weighted generalism score in plant communities

```
Family: gaussian  ( identity )
Formula:          genmod ~ prop.exotic + (1 | Community)
Data: mesoMeta
     AIC      BIC   logLik deviance df.resid
   514.6    526.8   -253.3    506.6      151
Random effects:
Conditional model:
 Groups    Name        Variance Std.Dev.
 Community (Intercept) 0.6338   0.7961
 Residual              1.2525   1.1191
Number of obs: 155, groups:  Community, 20
Dispersion estimate for gaussian family (sigma^2): 1.25
Conditional model:
            Estimate Std. Error z value Pr(>|z|)
(Intercept)   3.0673     0.3448   8.897  < 2e-16 ***
prop.exotic   2.1542     0.5667   3.802 0.000144 ***
Signif. codes:  0 '***' 0.001 '**' 0.01 '*' 0.05 '.' 0.1 ' ' 1
```

Table S1 List of all plant species used in the initial experiment and their provenance, functional group, mycorrhizal status, and nitrogen fixation status. Where very sparse evidence suggested a highly unusual mycorrhizal status for a particular group, we have inferred mycorrhizal status based on the weight of the evidence following a phylogeny-based classification of mycorrhizal types rather than a solely database driven approach

Plant species	Provenance	Functional group	Mycorrhizal types	Citation / method	Nitrogen fixing
Acacia dealbata	Exotic	Tree	AMF+EMF	(Warcup, 1980; Guisande-Collazo *et al.*, 2016)	Yes
Acaena caesiiglauca	Native	Forb	AMF	(Conner, 1981)	No
Acaena purpurea	Native	Forb	AMF	Visual	No
Achillea millefolium	Exotic	Forb	AMF	Visual	No
Agrostis capillaris	Exotic	Grass	AMF	(Gollotte *et al.*, 2004)	No
Alnus glutinosa	Exotic	Tree	EMF	(Thiem *et al.*, 2018)	Yes
Anemanthele lessoniana	Native	Grass	AMF	Inferred (Poaceae)	No

Anthoxanthum odoratum	Exotic	Grass	AMF	(Horton *et al.*, 2023)	No
Brachyglottis greyi	Native	Forb	AMF	Visual	No
Carex secta	Native	Grass	NM	(Meng *et al.*, 2023)	No
Chionochloa conspicua	Native	Grass	AMF	Inferred other Chionochloa	No
Cirsium vulgare	Exotic	Forb	AMF	Visual	No
Coprosma robusta	Native	Shrub	AMF	Visual and (Johnson, 1977)	No
Dactylis glomerata	Exotic	Grass	AMF	(West, 1996)	No
Echium vulgare	Exotic	Forb	AMF	Visual	No
Festuca novaezealandiae	Native	Grass	AMF	Visual	No
Holcus lanatus	Exotic	Grass	AMF	(West, 1996)	No
Hypericum perforatum	Exotic	Forb	AMF	Visual	No
Leptospermum scoparium	Native	Shrub	AMF+EMF	(Ramana *et al.*, 2023), (Weijtmans *et al.*, 2007)	No
Lolium perenne	Exotic	Grass	AMF	(Gollotte *et al.*, 2004)	No
Lupinus arboreus	Exotic	Forb	NM	(Oba *et al.*, 2001)	Yes
Medicago sativa	Exotic	Forb	AMF	(Campanelli *et al.*, 2013)	Yes
Muehlenbeckia astonii	Native	Shrub	NM	Inferred (Polygonaceae)	No

Muehlenbeckia complexa	Native	Shrub	NM	Inferred (Polygonaceae)	No
Olearia virgata	Native	Shrub	AMF	Inferred (likely)	No
Ozothamnus leptophyllus	Native	Forb	AMF	Visual	No
Phormium cookianum	Native	Forb	AMF	Visual	No
Pinus contorta	Exotic	Tree	EMF	(Dickie *et al.*, 2010)	No
Pinus radiata	Exotic	Tree	EMF	(Duñabeitia *et al.*, 2004)	No
Poa cita	Native	Grass	AMF	(Ramana *et al.*, 2023)	No
Poa colensoi	Native	Grass	AMF	(Powell, 1973)	No
Podocarpus totara	Native	Tree	AMF	Visual	No
Rumex acetosella	Exotic	Forb	NM	(Sandberg *et al.*, 2009)	No
Rumex obtusifolius	Exotic	Forb	NM	(Cavers & Harper, 1964; Zaller, 2004)	No
Sophora microphylla	Native	Shrub	AMF	(Ramana, 2022)	Yes
Trifolium pratense	Exotic	Forb	AMF	(Takács *et al.*, 2006)	Yes
Trifolium repens	Exotic	Forb	AMF	(Eason *et al.*, 2001)	Yes
Ulex europaeus	Exotic	Shrub	AMF	Visual and (Ramana *et al.*, 2023)	Yes
Veronica odora	Native	Shrub	AMF	Visual and (Ramana *et al.*, 2023)	No

Table S2 Subset of the original list of plant species known to associate with arbuscular mycorrhizal fungi and used in our analysis, along with each plant species provenance, functional group, nitrogen fixation status, and 1st 2nd and 3rd order root diameters.

Plant species	Provenance	Functional group	Nitrogen fixing	First order root diameter (mm)	Second_ order root diameter (mm)	Third order root diameter (mm)	Mean root diameter (mm)
Acacia dealbata	Exotic	Tree	Yes	0.255375	0.398	1.04083333	0.56473611
Acaena caesiiglauca	Native	Forb	No	0.1885	0.2145	0.247	0.21666667
Acaena purpurea	Native	Forb	No	0.1	0.1425	0.25	0.16416667
Achillea millefolium	Exotic	Forb	No	0.160625	0.234125	0.77575	0.39016667
Agrostis capillaris	Exotic	Grass	No	0.0715	0.1495	0.4745	0.23183333
Anemanthele lessoniana	Native	Grass	No	0.03333333	0.07	0.16333333	0.08888889
Anthoxanthum odoratum	Exotic	Grass	No	0.1404	0.195	0.3042	0.2132
Brachyglottis greyi	Native	Forb	No	0.40966667	0.502	0.928	0.61322222
Chionochloa conspicua	Native	Grass	No	0.048	0.0675	0.24	0.1185
Cirsium vulgare	Exotic	Forb	No	0.23916667	0.36116667	0.617625	0.40598611
Coprosma robusta	Native	Shrub	No	0.197625	0.254125	0.38525	0.279
Dactylis glomerata	Exotic	Grass	No	0.02	0.03333333	0.08666667	0.04666667
Echium vulgare	Exotic	Forb	No	0.16	0.395	1.625	0.72666667
Festuca novaezealandiae	Native	Grass	No	0.11	0.11	0.215	0.145
Holcus lanatus	Exotic	Grass	No	0.105	0.13	0.45	0.22833333
Hypericum perforatum	Exotic	Forb	No	0.175	0.26011111	0.4598125	0.29830787
Leptospermum scoparium	Native	Shrub	No	0.2668	0.3115	0.41275	0.33035
Lolium perenne	Exotic	Grass	No	0.035	0.075	0.155	0.08833333

Medicago sativa	Exotic	Forb	Yes	0.0775	0.14666667	0.48333333	0.23583333
Olearia virgata	Native	Shrub	No	0.23233333	0.62933333	0.67	0.51055556
Ozothamnus leptophyllus	Native	Forb	No	0.273	0.49966667	0.702	0.49155556
Phormium cookianum	Native	Forb	No	0.22666667	0.14333333	0.87666667	0.41555556
Poa cita	Native	Grass	No	0.04	0.09	0.3	0.14333333
Poa colensoi	Native	Grass	No	0.1625	0.20475	0.356375	0.24120833
Podocarpus totara	Native	Tree	No	0.51108333	0.69466667	0.80925	0.67166667
Sophora microphylla	Native	Shrub	Yes	0.468	0.638625	0.4705	0.52570833
Trifolium pratense	Exotic	Forb	Yes	0.191125	0.2915	0.500875	0.32783333
Trifolium repens	Exotic	Forb	Yes	0.19	0.2	0.35	0.24666667
Ulex europaeus	Exotic	Shrub	Yes	0.305	0.345	0.5	0.38333333
Veronica odora	Native	Shrub	No	0.39	0.41	0.48	0.42666667

References

Campanelli A, Ruta C, De Mastro G, Morone-Fortunato I. **2013**. The role of arbuscular mycorrhizal fungi in alleviating salt stress in Medicago sativa L. var. icon. *Symbiosis* **59**: 65–76.
Cavers PB, Harper JL. **1964**. Rumex Obtusifolius L. and R. Crispus L. *Journal of Ecology* **52**: 737–766.
Conner LN. **1981**. Ecophysiology of five subalpine Acaena species in relation to habitat.
Dickie IA, Bolstridge N, Cooper JA, Peltzer DA. **2010**. Co-invasion by Pinus and its mycorrhizal fungi. *New Phytologist* **187**: 475–484.
Duñabeitia MK, Hormilla S, Garcia-Plazaola JI, Txarterina K, Arteche U, Becerril JM. **2004**. Differential responses of three fungal species to environmental factors and their role in the mycorrhization of Pinus radiata D. Don. *Mycorrhiza* **14**: 11–18.
Eason WR, Webb KJ, Michaelson-Yeates TPT, Abberton MT, Griffith GW, Culshaw CM, Hooker JE, Dhanoa MS. **2001**. Effect of genotype of Trifolium repens on mycorrhizal symbiosis with Glomus mosseae. *The Journal of Agricultural Science* **137**: 27–36.
Gollotte A, van Tuinen D, Atkinson D. **2004**. Diversity of arbuscular mycorrhizal fungi colonising roots of the grass species Agrostis capillaris and Lolium perenne in a field experiment. *Mycorrhiza* **14**: 111–117.
Guisande-Collazo A, González L, Souza-Alonso P. **2016**. Impact of an invasive nitrogen-fixing tree on arbuscular mycorrhizal fungi and the development of native species. *AoB PLANTS* **8**: plw018.
Horton S, Lowther ,Mandy, and Lord J. **2023**. Anthoxanthum odoratum (Poaceae) as a bioindicator of arbuscular mycorrhizal fungi abundance prior to native revegetation on agricultural soils. *New Zealand Journal of Botany* **0**: 1–11.
Johnson PN. **1977**. Mycorrhizal Endogonaceae in a New Zealand Forest. *New Phytologist* **78**: 161–170.
Kowal J, Arrigoni E, Lane S. **2020**. Acidified Blue Ink-staining Procedure for the Observation of Fungal Structures Inside Roots of Two Disparate Plant Lineages. *Bio-protocol* **10**: e3786.
Oba H, Tawaray K, Wagatsuma T. **2001**. Arbuscular mycorrhizal colonization in *Lupinus* and related genera. *Soil Science and Plant Nutrition* **47**: 685–694.
Powell CL. **1973**. Mycorrhizal status of rushes and sedges in New Zealand.
Ramana J. **2022**. Native and exotic plant root traits and arbuscular mycorrhizal fungal community composition and function.
Ramana JV, Tylianakis JM, Ridgway HJ, Dickie IA. **2023**. Root diameter, host specificity and arbuscular mycorrhizal fungal community composition among native and exotic plant species. *New Phytologist* **239**: 301–310.
Sandberg J, Jones DL, Fransson A-M. **2009**. Despite high uptake efficiency, non-mycorrhizal Rumex acetosella increases available phosphorous in the rhizosphere soil, whereas Viscaria vulgaris, Plantago lanceolata and Achillea millefolium does not. *Nordic Journal of Botany* **27**: 444–448.
Takács T, Osztoics ,Erzsébet, Csathó ,Péter, Csillag ,Julianna, Rajkai-Végh ,Krisztina, Magyar ,Marianna, and Lukács A. **2006**. Comparative Effects of Rock Phosphates on Arbuscular Mycorrhizal Colonization of Trifolium pratense L. *Communications in Soil Science and Plant Analysis* **37**: 2779–2790.
Thiem D, Piernik A, Hrynkiewicz K. **2018**. Ectomycorrhizal and endophytic fungi associated with Alnus glutinosa growing in a saline area of central Poland. *Symbiosis* **75**: 17–28.
Warcup J h. 1980. Ectomycorrhizal Associations of Australian Indigenous Plants. *New Phytologist* **85**: 531–535.

Weijtmans K, Davis M, Clinton P, Kuyper TW, Greenfield L. **2007**. Occurrence of arbuscular mycorrhiza and ectomycorrhiza on Leptospermum scoparium from the Rakaia catchment, Canterbury. *New Zealand Journal of Ecology* **31**: 255–260.
West HM. **1996**. Influence of Arbuscular Mycorrhizal Infection on Competition between Holcus Lanatus and Dactylis Glomerata. *Journal of Ecology* **84**: 429–438.
Zaller JG. **2004**. Ecology and non-chemical control of Rumex crispus and R. obtusifolius (Polygonaceae): a review. *Weed Research* **44**: 414–432.
